# Proposed revision of the 8th edition AJCC clinical staging system for esophageal squamous cell cancer treated with definitive chemo-IMRT based on CT imaging

**DOI:** 10.1186/s13014-019-1258-4

**Published:** 2019-03-28

**Authors:** Mingqiu Chen, Xiqing Li, Yuangui Chen, Pingping Liu, Zhiwei Chen, Minmin Shen, Xiaohong Liu, Yu Lin, Rongqiang Yang, Wei Ni, Xin Zhou, Lurong Zhang, Ye Tian, Junqiang Chen, Lengxi Fu

**Affiliations:** 1 Department of Radiation Oncology, Fujian Cancer Hospital & Fujian Medical University Cancer Hospital, No. 420, Fumalu Road, Jinan District, Fuzhou City, Fujian Province People’s Republic of China 350014; 2 Fujian Provincial Platform for Medical Laboratory Research of First Affiliated Hospital, Fuzhou, Fujian People’s Republic of China; 3 The Graduate School, Fujian Medical University, Fuzhou, Fujian People’s Republic of China; 4 Department of Radiation Oncology, Fujian Medical University Union Hospital, Fuzhou, Fujian People’s Republic of China; 5 Fuzhou Center for Disease Control and Prevention, Fuzhou, Fujian People’s Republic of China; 6 Cancer and Genetics Research Complex, Department of Molecular Genetics and Microbiology, College of Medicine, University of Florida, Gainesville, FL USA; 7 Department of Radiation Oncology, the Second Affiliated Hospital of Soochow University, Suzhou, Jiangsu China; 8 Institute of Radiotherapy & Oncology, Soochow University, Suzhou, Jiangsu China; 9 Central Laboratory, the First Affiliated Hospital of Fujian Medical University, Fuzhou City, Fujian Province People’s Republic of China 350005

**Keywords:** Concurrent chemoradiotherapy, Esophageal squamous cell carcinoma, Staging, Survival

## Abstract

**Purpose:**

To validate and propose revision of the 8th edition American Joint Committee on Cancer (AJCC) clinical staging system for esophageal squamous cell cancer (ESCC) patients treated with definitive intensity-modulated radiation therapy combined with concurrent chemotherapy (Chemo-IMRT) based on computed tomography (CT) imaging.

**Methods:**

The clinical data of patients with ESCC treated with Chemo-IMRT were collected and retrospectively reviewed. All CT images were independently reevaluated and restaged according to the 8th edition AJCC staging system. The overall survival (OS) rates were analyzed statistically. ROC curves of the various parameters of the primary tumor and metastatic lymph nodes were generated in order to identify the cutoff values correlated to patient survival using the area under curve.

**Results:**

The gross tumor volume of the primary tumor (GTV-prT) and the clinical N stage (cN) were independent factors that influenced OS. The 5-year OS rate of patients with GTV-prT ≤28 cm^3^, GTV-prT > 28 and ≤ 56 cm^3^, and GTV-prT > 56 cm^3^ were 54.6, 31.1 and 18.6%, respectively. The 5-year OS rate of patients with cN0, cN1 SLNM (−), cN2 SLNM (−), cN3 SLNM (−) and SLNM (+) were 62.8 (*P* < 0.001), 34.0 (*P* = 0.16), 20.0 (*P* = 0.785), 0 (P < 0.001) and 26.9%, respectively. After restaging the SLNM as regional MLNs, the 5-year OS rates of the patients with cN0, 1, 2 and 3 were 62.8, 36.3, 23.7 and 7.8%, respectively. Various GTV-prT were combined with the cN to establish a new clinical TNM staging system: I, GTV-prT1 and cN0; II, GTV-prT2 or 3 and cN0, GTV-prT1 and cN1; III, GTV-prT1 and cN2, GTV-prT2 and cN1,2; Iva, GTV-prT3 and cN1,2; IVb, GTV-prT_any_ and cN3; IVc, T_any_N_any_M1. Subsequently, the OS differed significantly between the adjacent GTV-prT cN categories, except those of stage I vs. II.

**Conclusion:**

The SLNM should be dealt with as a regional rather than a distant disease in patients with ESCC when treated with CRT. The proposed nonsurgical staging system based on the GTV-prT and N appears to be a simple and accurate prognosis predictor for patients with ESCC who have undergone definitive Chemo-IMRT.

## Introduction

Esophageal cancer (EC) is a common type of cancer, with the 8th highest incidence and the 6th highest cause of cancer-related mortalities worldwide [1, 2]. Surgery has a cornerstone role in the treatment of patients with EC [3, 4]. However, due to their physiological conditions, the tumor location or the tumor stage, only approximately 25% of newly diagnosed patients are eligible for surgery [5]. For patients with unresectable EC, definitive chemoradiation therapy (CRT) is considered to be the optimal treatment [6].

As part of the continuous advancements in radiotherapy equipment and techniques, intensity-modulated radiotherapy (IMRT) technology has been applied in clinic and confirmed to provide improved tumor target coverage and better sparing of normal structures compared to two-dimensional conventional radiotherapy or three-dimensional conformal RT [7]. IMRT combining with concurrent chemotherapy (Chemo-IMRT) of EC has been shown to reduce radiation-induced toxicities, enhance local control and improve long-term survival [8]. However, due to the lack of an accurate staging system in predicting prognosis of EC patients treated with radiotherapy, it is difficult to evaluate the efficacy of Chemo-IMRT and communicate the treatment experience to each other without ambiguity.

Although several organizations have proposed staging systems to predict the prognosis of patients with EC [9, 10], only the American Joint Committee on Cancer (AJCC) TNM (tumor-node-metastasis) staging system is globally understood and universally accepted. The latest edition, the 8th AJCC staging system [11], provides a clinical staging system of EC for treatment decision-making and prognosis prediction based on pre-surgery imaging for the first time. However, as it is the first version and it requires efficient resolution of imaging methods, there are few studies that verify the prediction accuracy and discriminatory ability of the 8th AJCC staging system for EC treated with radiotherapy, especially with IMRT [12].

Compared to computed tomography (CT), an endoscopic ultrasound (EUS) is currently considered to be the most accurate imaging modality for early T and N staging in patients with EC [13]. However, the majority of patients with ESCC commonly present with stenotic tumors, which may prevent the passage of the endoscope and evaluation of the entire tumor and distal esophagus, attenuating the value of an EUS in staging EC [14]. To validate the efficacy of clinical staging in patients with EC that were treated with CRT based on CT or PET-CT imaging plays a more valuable role in treatment decisions, prognosis prediction and research.

In the present study, clinical data from patients with esophageal squamous cell cancer (ESCC) that were treated with Chemo-IMRT based on CT imaging were collected retrospectively and analyzed in order to assess the applicability of the 8th edition AJCC clinical staging system.

## Patients and methods

### Patient selection criteria

The current study was approved by Fujian Cancer Hospital & Fujian Medical University Cancer Hospital Institutional Review Board (No. K201427). The patients treated at Fujian Cancer Hospital & Fujian Medical University Cancer Hospital who fulfilled the following inclusion criteria were selected [15, 16]: histologically confirmed ESCC with no distant metastases except for metastatic supraclavicular lymph nodes (SLNMs); ≤70 years old; Eastern Cooperative Oncology Group scoring ≤2; pretreatment CT or PET-CT images available for defining the clinical stage; pretreatment standard laboratory tests available to assess the adaptation for chemotherapy and RT; treated initially with definitive chemo-IMRT without salvage surgery; and sufficient follow-up data available for survival assessment.

### Treatment

All patients enrolled in the current study were treated initially with Chemo-IMRT. The details of the IMRT, including gross tumor volume (GTV), clinical target volume and organs at risk (OAR) of radiotherapy, target doses and dose limitations of the OAR, were defined and adjusted as described in our previous study [12, 15]. All patients were treated with concurrent platinum- or taxane-based chemotherapy with or without neoadjuvant or adjuvant chemotherapy.

### Clinical staging

The CT scanning imaging data were revaluated independently by two radiologists that specialize in thoracic cancer. Any disagreements were resolved by consensus. The clinical records and CT results of all patients were collated into a database in order to restage the patients accurately according to the criteria of the 8th edition AJCC staging system.

### Surveillance

The follow-up schedule for the patients was as previously reported [15]. In brief, patients were evaluated every 3 months for the first 2 years after CRT, every 6 months for the next 3 years, and then once annually. All patient outcomes were evaluated in July 2018.

### Statistical analysis

Data were analyzed using SPSS, version 24.0 (IBM Corp., Armonk, NY, USA). The overall survival (OS), which was the primary endpoint of the current study, was measured from the day of diagnosis to the date of mortality or last follow-up. Survival curves were produced using the Kaplan-Meier estimator method and compared using the log-rank test. Multivariable analysis of the clinical baseline characteristics was performed using the Cox proportional hazards model and included gender, age, tumor location, maximum diameter of the primary tumor (D_max-_prT), length of the primary tumor (L-prT), GTV of the primary tumor (GTV-prT), clinical T stage (cT), maximum diameter of the metastatic lymph nodes (MLN; D_max_-MLNs), total GTV of the MLNs (tGTV-MLNs), SLNM, clinical N stage (cN) and clinical TNM group (cTNM). ROC curves of the various parameters of the primary tumor and MLNs were generated in order to identify the cutoff value correlated to patient survival using the area under the curve. The confidence intervals represent the 95% lower and upper bounds. *P* ≤ 0.05 was considered to indicate a statistically significant difference.

## Results

### Patient characteristics

Between March 1, 2010 and December 31, 2016, a total of 356 patients treated with definitive Chemo-IMRT at Fujian Cancer Hospital & Fujian Medical University Cancer Hospital met the inclusion criteria and their data were collected for analysis. The characteristics of the patients are presented in Table 1.

**Table 1:**
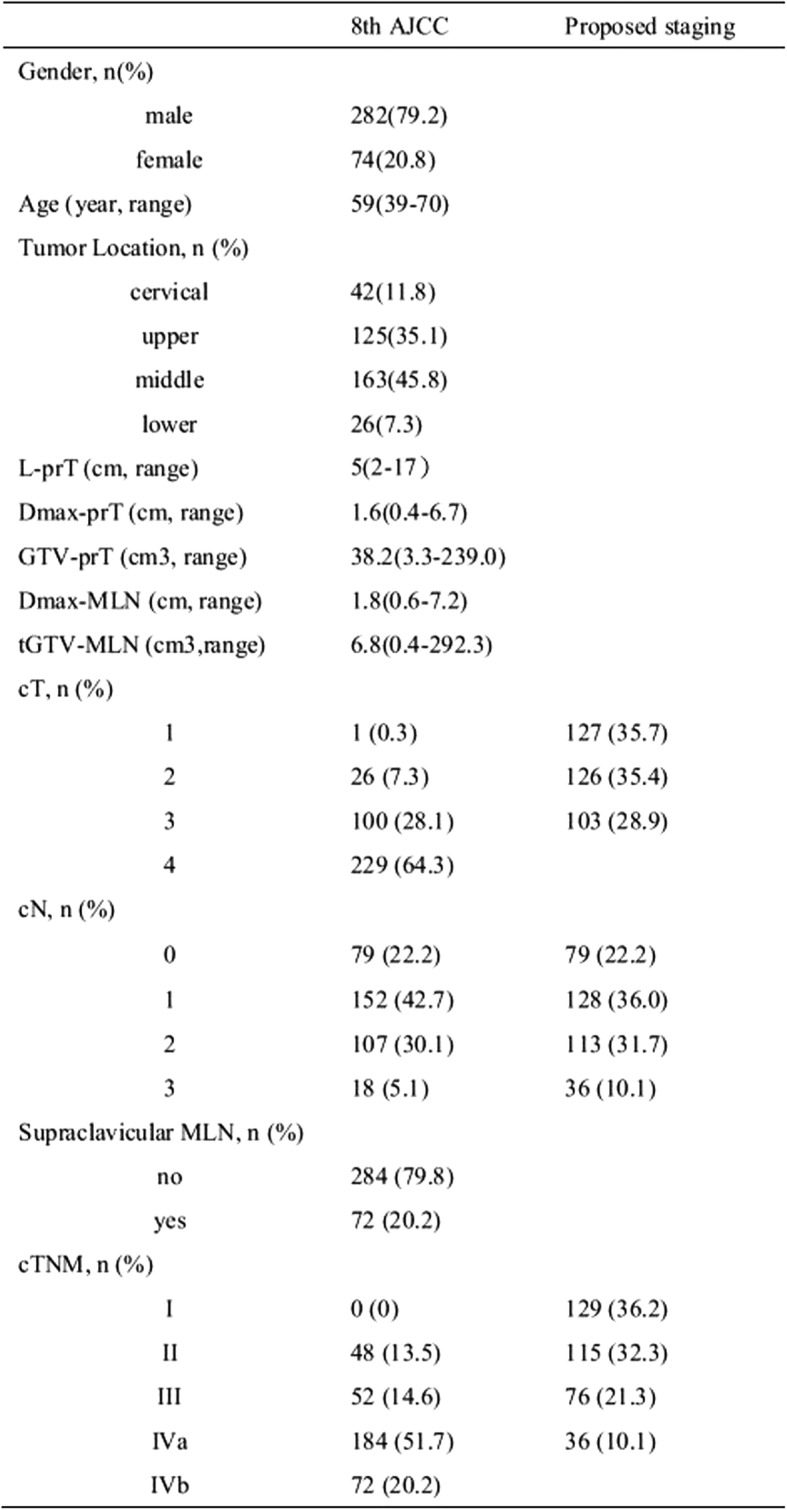
Clinical characteristics of the patients

### Survival and patterns of treatment failure

At the last follow-up appointment, a total of 137 patients were alive and 219 patients had died. Among the mortalities, 151 patients had succumbed to the disease (50, primary tumor; 29, local regional node metastases; 61, distant metastases; and 13, both a primary tumor and distant metastases), 20 had succumbed to a non-tumor disease and 46 patients had died due to unknown reasons.

The median follow-up times of the entire cohort patients and those that had survived were 23.9 (2.4–129.2) and 48.5 (20.6–129.2) months, respectively. The 1, 3 and 5-year OS rates were 78.7, 43.6 and 35.6%, respectively (Table 2). The univariate and multivariate analyses indicated that the GTV-prT and cN were significant independent factors that influenced OS (*P* < 0.001) (Table 3).

**Table 2:**
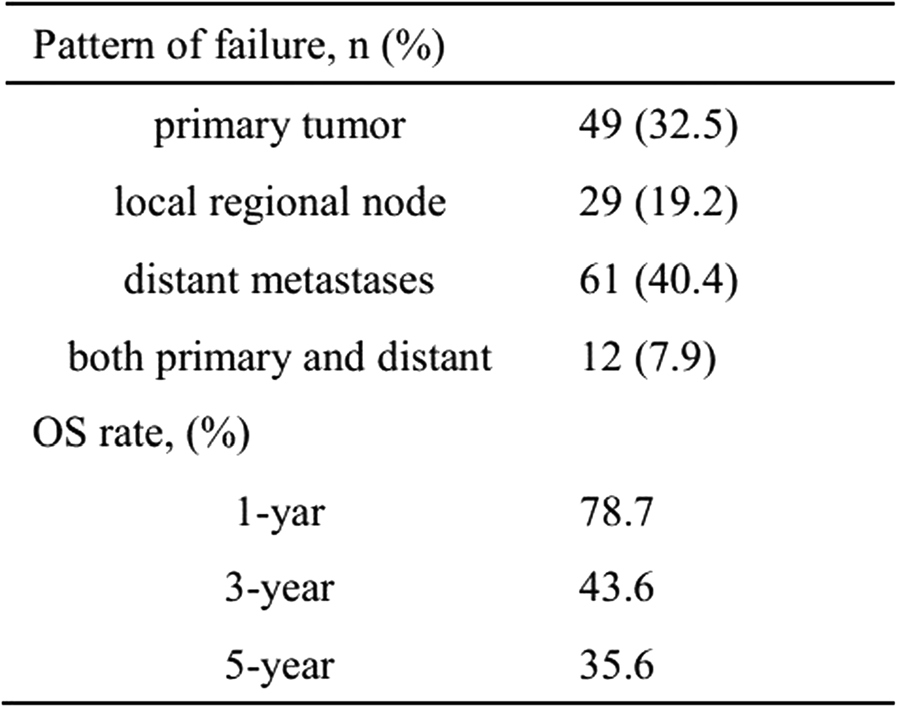
Failure pattern and survival

**Table 3:**
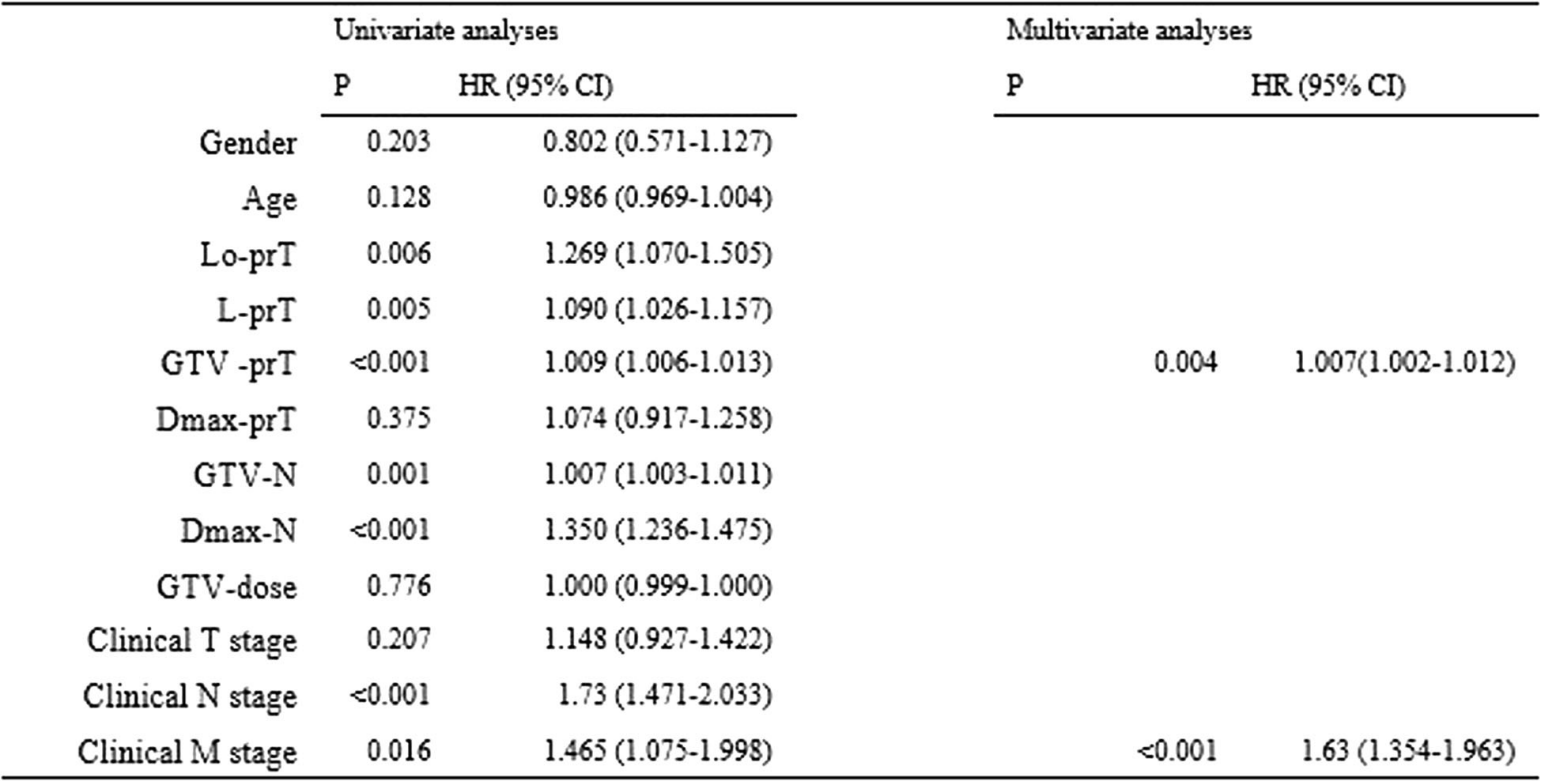
Prognostic factors by univariate and multivariate analyses

### Validation and proposed revision of the 8th edition AJCC clinical staging system

#### T categories

**Fig. 1:**
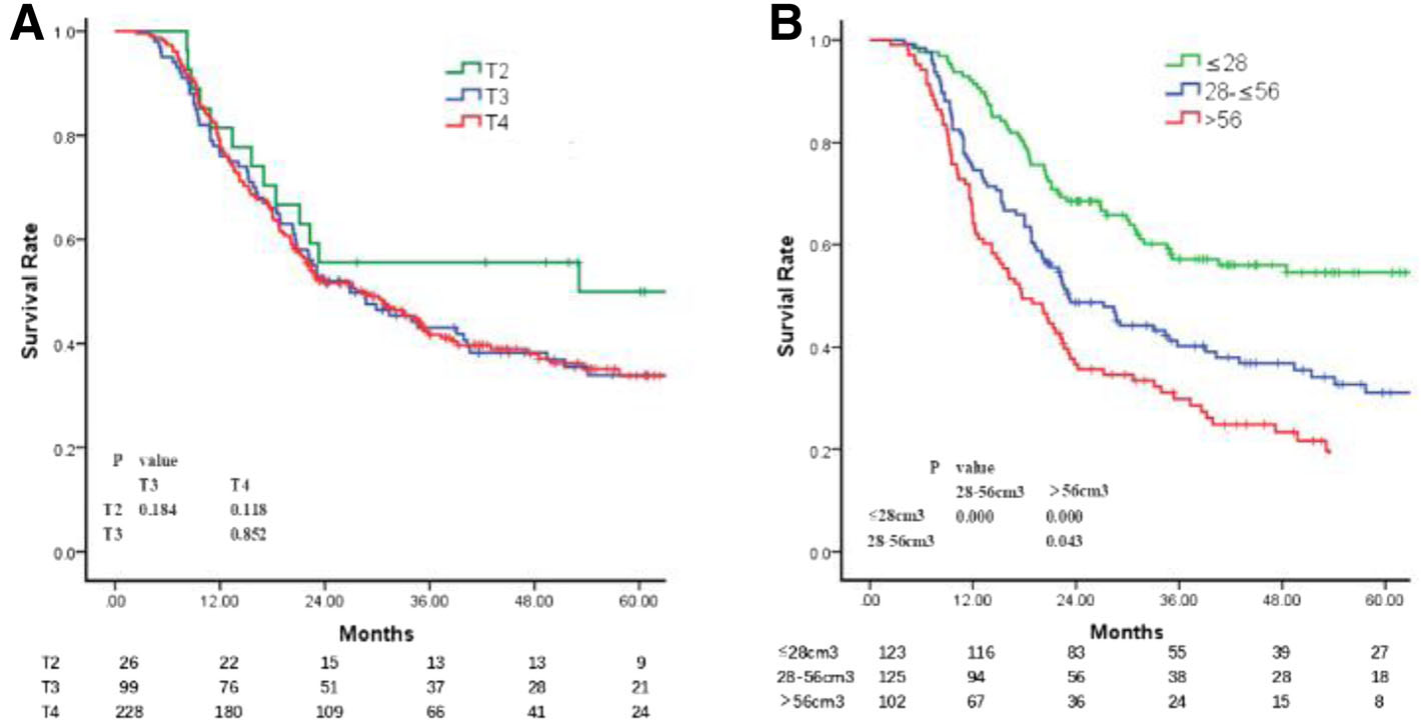
**a** Overall survival of patients with different clinical T stages. **b**: Overall survival of patients with GTV-prT1, GTV-prT2 and GTV-prT3

As there was only one patient with cT1 disease in the current study, those with cT1 and cT2 were combined for the further analysis. The 5-year OS rates of the patients with cT2 (cT1 + cT2), cT3 and cT4 disease were 50.0, 33.6 and 34.5%, respectively. The differences in the OS among the different cT subgroups or between adjacent cT subgroups (cT2 vs. cT3 and cT3 vs. cT4) were not significant (Fig. 1a).

As the GTV-prT was an independent factor that influenced the OS, a ROC curve was generated to determine the cutoff value of the GTV-prT in survival and it indicated that the survival in the subgroups differed significantly with the cutoffs at 28 and 56 cm^3^. The 5-year OS rates of the patients in the GTV-prT ≤28 cm^3^, GTV-prT > 28 and ≤ 56 cm^3^ and GTV-prT > 56 cm^3^ subgroups were 54.6, 31.1 and 18.6%, respectively. The differences between the adjacent GTV-prT subgroups were significant (Fig. 1b). Therefore, we recommend that the cT is subdivided into GTV-prT1, GTV-prT2 and GTV-prT3 based on the GTV-prT ≤28 cm^3^, GTV-prT > 28 and ≤ 56 cm^3^ and GTV-prT > 56 cm^3^ subgroups in order to predict survival.

### SLNM and N categories

**Fig. 2:**
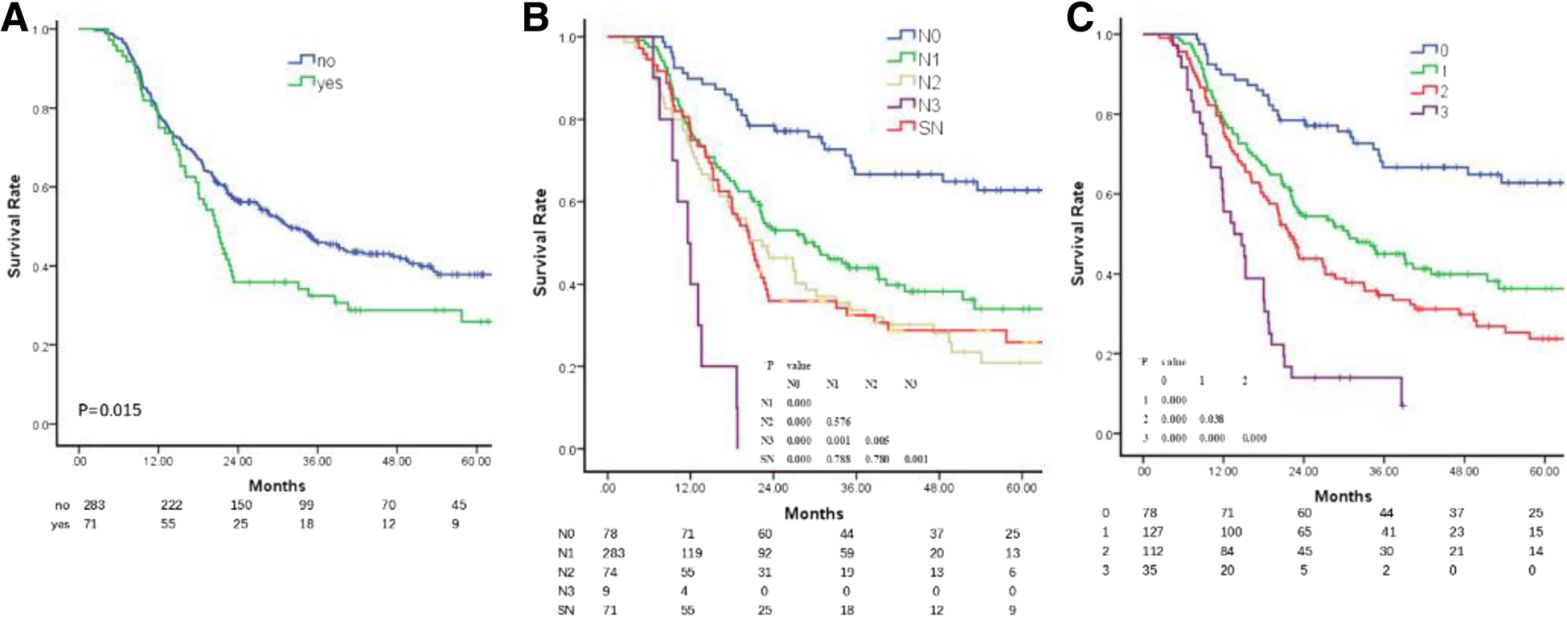
**a** Overall survival of patients with supraclavicular lymph node metastasis. **b** Overall survival of patients with different clinical N stages and supraclavicular lymph node metastasis. **c**: Overall survival of patients with different clinical N stages when metastatic supraclavicular lymph nodes were considered as regional metastatic lymph nodes

The univariate, but not the multivariate, Cox proportional hazards model analyses indicated that SLNM was an independent risk factor for the OS. Patients with SLNM (−) had a higher survival rate to those with SLNM (+) (5-year OS, 37.8 and 26.9%, respectively; *P* = 0.034) (Fig. 2a).

Survival analysis was conducted considering the SLNM as distant metastatic disease, and the 5-year OS rates of the patients with cN0 with SLNM (−), cN1 with SLNM (−), cN2 with SLNM (−), cN3 with SLNM (−), and SLNM (+) with cN1 or cN2 or cN3 were 62.8, 34.0, 20.0, 0 and 26.9%, respectively. The OS of the patients with SLNM (+) was lower compared to those with cN0 with SLNM (−) (*P* < 0.001), but higher compared to those with cN3 with SLNM (−) (P < 0.001) and similar to those with cN1 with SLNM (−) (*P* = 0.16) or cN2 with SLNM (−) (0.785) (Fig. 2b). Based on these results, SLNMs were considered as MLNs and subsequent survival analysis was conducted.

The clinical characters of the MLNs, including the D_max_-MLNs, tGTV-MLNs and cN, were collected for analyzing the independent factors for survival by multivariate Cox hazards model analyses. The results indicated that cN was the only significant influencing factor of OS; as the cN increased, the survival rate deteriorated. The 5-year OS rates in the cN0, cN1, cN2 and cN3 groups were 62.8, 36.3, 23.7 and 7.8%, respectively. The survival differences among the patients with various cN were significant (Fig. 2c).

### Stage group

**Fig. 3:**
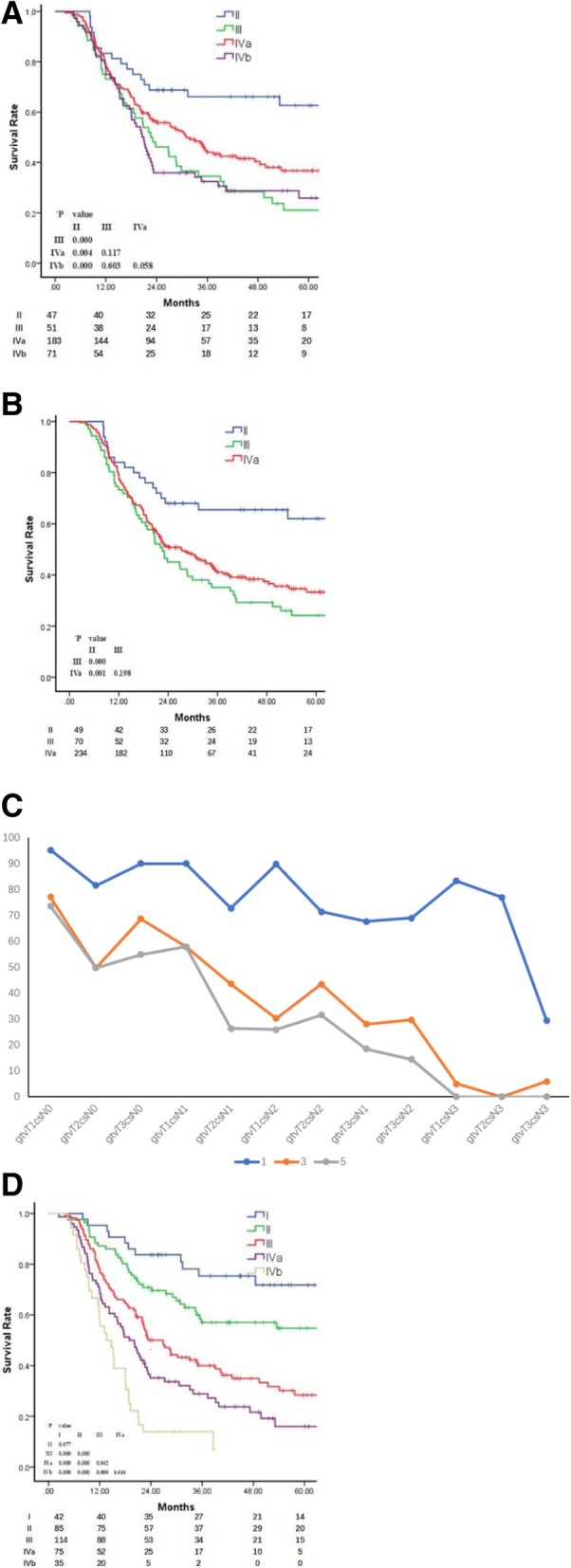
**a** Overall survival of patients with supraclavicular lymph node metastasis considered as distant metastasis, as per the 8th AJCC TNM staging system. **b**: Overall survival of patients with metastatic supraclavicular lymph nodes as regional metastatic lymph nodes, as per the 8th AJCC TNM staging system. **c**: Proposed subgroups based on the 1, 3 and 5-year overall survival rates. **d**: Overall survival of patients according to the proposed staging system based on the GTV-prTcN

According to the 8th AJCC clinical stage, the 5-year OS rates of the enrolled patients with stages II, III, IVa and IVb (with metastatic SLNM) were 62.0, 24.5, 35.8 and 25.1%, respectively. The survival of those with stage II was higher compared to stages III, IVa and IVb (*P* < 0.05). The differences among the latter three subgroups were not significant (Fig. 3a).

When analyzing the stages with SLNMs as regional MLNs, the 5-year OS rates of the patients with stages II, III and IVa were 62.0, 24.1 and 32%, respectively. The survival rate of the patients with stage II was higher compared to stages III and IVa (P < 0.05), whereas the difference in the rate between stages III and IVa was not significant (*P* = 0.25) (Fig. 3b).

In order to investigate a rational TNM grouping system that distinctly predicts the prognosis of patients, a proposed TNM combination system based on the GTV-prT subgroups and cN subgroups were analyzed using the Cox hazards model, with each subset represented by an indicator variable and T1 N0 as the reference group. The proposed groups are as follows: I, GTV-prT1 and cN0; II, GTV-prT2 or 3 and cN0, GTV-prT1 and cN1; III, GTV-prT1 and cN2, GTV-prT2 and cN1,2; IVa, GTV-prT3 and cN1,2; IVb, GTV-prT_any_ and cN3; and IVc, T_any_N_any_M1 (with distant metastasis but not SLNMs). The results demonstrated that when the GTV-prTcN categories were considered as the four groups (Fig. 3c), the OS rates differed significantly between the adjacent GTV-prTcN categories, except for the I and II groups, which may be due to the small sample size in the current study (Fig. 3d).

## Discussion

The T category of the current AJCC TNM staging system for EC is based on tumor invasion of the esophageal wall. The extent of local tumor invasion determines the potential for radical resection of the tumor and ultimately determines the prognosis of patients. Patients with a higher T category are associated with a lower OS rate. However, in the current study, the survival rate among the various cT subgroups did not differ significantly, indicating that the current cT staging based on CT imaging is not sufficient in accurately predicting the prognosis of patients treated with Chemo-IMRT. A reasonable explanation is that, unlike surgery that is subject to local tumor invasion, patients who receive radiotherapy with modern technology such as IMRT can achieve perfect primary tumor dose coverage and improve local control even in cases of wide-ranging local tumor invasion [7].

Numerous studies had been conducted to explore the correlation between the clinical parameters of the primary tumor, including the L-prT, Dmax-prT and GTV-prT, and the survival of patients with ESCC. To the best of our knowledge, there is no consensus concerning the optimal cut-off value to predict the OS rate [17–20] due to different sample sizes, different histological types, variable inclusion criteria and, most importantly, unreliable statistical methods used to determine the cut-off points [21]. Despite the paucity of convictive studies, there are cumulative studies that support the existence of a link between the GTV-prT and the outcome of patients with EC [22, 23]. Similarly, the current study indicated that only the GTV-prT, rather than the L-prT, Dmax-prT or cT stage, had a strong prognostic effect on OS. In addition, subclassification of the patients into different T stages according to the GTV-prT cut-off value demonstrated a superior ability in predicting the OS. Therefore, we recommend that the GTV-prT should be taken into consideration as a stage criterion to define the cT in patients treated with Chemo-IMRT.

Although several studies have shown that patients with tumors in the lower segment of the esophagus have a better prognosis because of the increased success of resection, the influence of the tumor location on OS differs. Li et al. reported that tumor location was an independent prognostic factor for patients with ESCC after esophagectomy [18], whereas Doki et al. reported that patients with different locations of EC had similar 5-year OS rates but different sites of tumor recurrence [17]. In the current study, the OS of patients with various tumor locations did not differ significantly. IMRT technology, which provides excellent dose coverage and conformity to the target volume, may account for the results [24, 25]. The result illimunates that tumor location is no longer an important factor affecting the prognosis of patients with ESCC when administered with Chemo-IMRT.

ESCC patients with SLNM are considered to have distant metastasis even in the newest 8th edition AJCC TNM staging system [26] and are reported with poorer prognoses. Recently, several studies have argued that SLNM does not constitute an important independent prognostic factor for patients treated with CRT [27]. The current study demonstrates that the OS of patients with SLNM(+) is higher compared to cN3 SLNM(−) and is similar to cN1 SLNM(−) or cN2 SLNM(−), but lower compared to cN0 SLNM(−), which indicates that patients with SLNMs should be considered to have regional MLNs and be treated with curative intent [28].

In our previous study, we found that the D_max_-MLN had the potential to predict survival; as the D_max_-MLN increased, the survival rate decreased. We proposed simplified N categories by utilizing the D_max_-MLN on CT imaging in complement with the current cN staging system in order to predict prognosis [12]. However, the current study did not reproduce our previous results and demonstrated that the D_max_-MLN has no correlation with the OS. The discrepancy between the current and previous studies may be due to the adverse D_max_-MLN influence on survival being minimized by the radiosensitization of synchronous chemotherapy.

As the number of MLNs is a vital prognostic factor for patients with EC undergoing R0 resection [19], the number of MLNs was used to determine the N categories in the AJCC staging system after the 7th edition, which has a good prognostic value for surgical patients. Similarly, in the current study, patients with different cN based on the number of MLNs achieved different survival rates. Furthermore, cN played a predominant risk proportion to the GTV-prT, although both the cN and GTV-prT were independent prognostic factors in the current study. Once patients developed cN3, the survival rate was markedly lower compared to the cN1 or cN2 stages, thus was not different among various GTV-prT subgroups. However, in the patients with cN1 or cN2, the GTV-prT clearly affected survival.

Based on these results, we recommend a simplified and easy-to-apply staging system to better predict the outcome of patients, based on various combinations of GTV-prT subgroups and cN subgroups, which has the following groups: I, GTV-prT1 and cN0; II, GTV-prT2 or 3 and cN0, GTV-prT1 and cN1; III, GTV-prT1 and cN2, GTV-prT2 and cN1,2; IVa, GTV-prT3 and cN1,2; IVb, GTV-prT_any_ and cN3; and IVc, T_any_N_any_M1.

However, due to limitations of the present study, including a single center and retrospective design, the suboptimal assessment of clinical TNM staging based on CT but not PET-CT and/or EUS [29], the diagnosis of SCLN only basing on the CT imagines but not histology, the small patient sample enrolled and the paucity of histologic grade analysis, multicenter studies with larger sample size will be required to validate this staging system.

## Conclusions

In conclusion, the current study confirms that SLNM should be considered as a regional, rather than distant, disease in patients with ESCC when treated with Chemo-IMRT. The proposed nonsurgical staging system based on the GTV-prT and N appears to be a simple and accurate prognosis predictor in those patients.
